# Food-Induced Duodenal Obstruction Successfully Reopened by Endoscopic Treatment

**DOI:** 10.7759/cureus.12176

**Published:** 2020-12-19

**Authors:** Satoshi Masuda, Taiki Aoyama, Akira Fukumoto, Shinji Nagata

**Affiliations:** 1 Gastroenterology, Hiroshima City Asa Citizens Hospital, Hiroshima, JPN

**Keywords:** double-balloon enteroscopy, duodenum, endoscopic treatment, food, ileus

## Abstract

Duodenal obstruction is a rare event that is unlikely to be treated endoscopically. Herein, we describe the case of a 75-year-old woman who presented with vomiting and was diagnosed with food-induced duodenal obstruction. Impacted food was fragmented and removed by double-balloon enteroscopy, and the duodenal tract was reopened without any adverse events. Follow-up capsule endoscopy was performed one month after treatment to determine the obstruction etiology and it revealed a remarkably delayed passage of the capsule through the duodenum and excessive amounts of floating food residue in the third portion of the duodenum. Obstruction recurrence was not observed six months after endoscopic treatment. In conclusion, in our case, endoscopic treatment of duodenal obstruction prevented the unnecessary performance of surgery, suggesting its clinical utility for this condition.

## Introduction

Management of an acquired duodenal obstruction is challenging because of the limited treatment options. Owing to the anatomical complexity of the area, even minimally invasive surgical procedures, such as pancreas-sparing or segmental resection of the duodenum, are associated with an 11.8%-38% rate of adverse events, including duodenal leak, fistulas and hemorrhage [1-3]. Transluminal endoscopic treatment could be a viable option as endoscopic treatment is already an important alternative to surgery for cases of hemostasis and removal of a foreign body from the small bowel [4-6].

In this report, we describe a case of food-induced mechanical obstruction in the third portion of the duodenum. Endoscopic treatment was selected instead of surgery to reopen the obstruction.

## Case presentation

**Figure 1:**
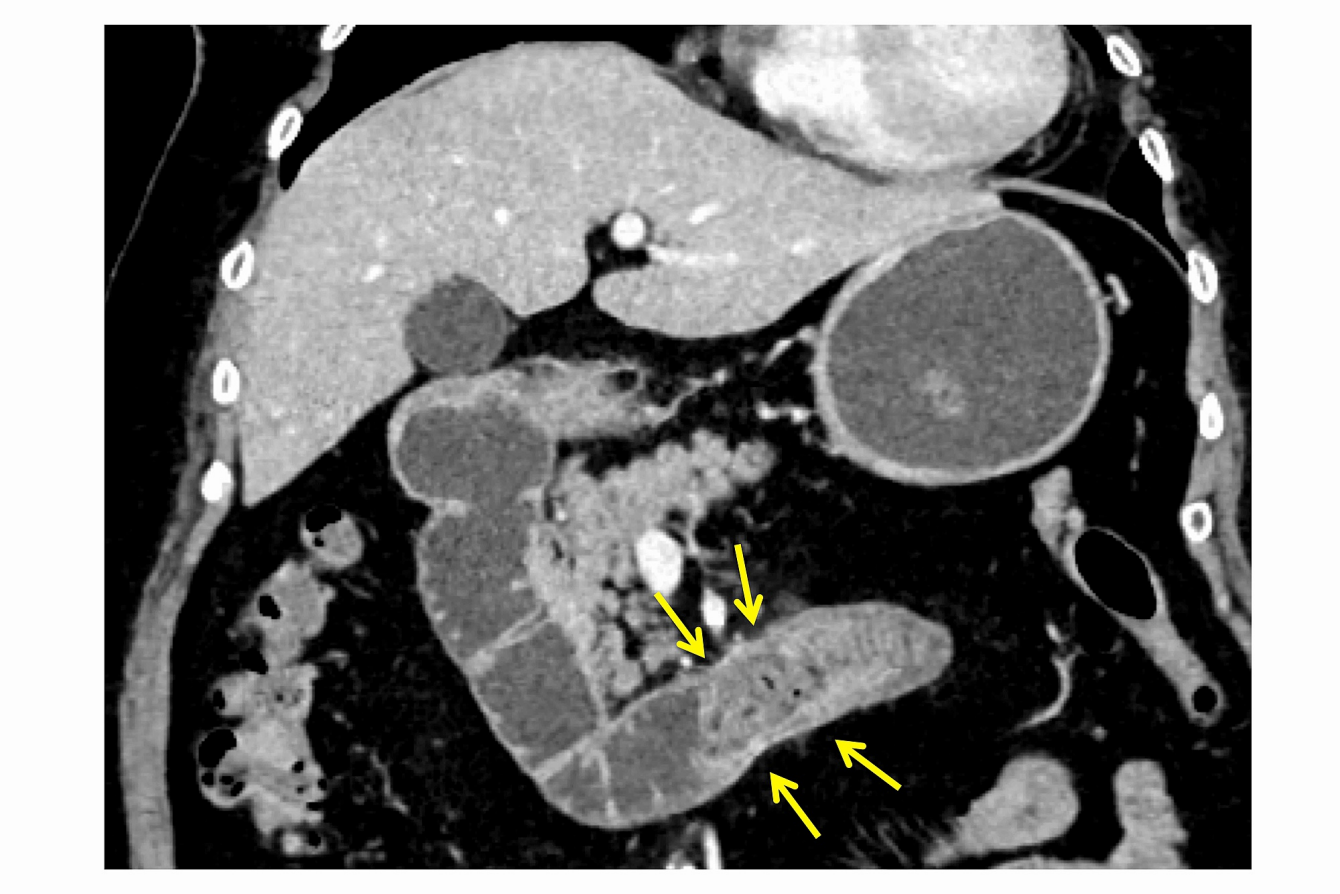
Abdominal computed tomography at admission Bowel obstruction is seen in the third portion of the duodenum (yellow arrows).

A 75-year-old woman with no history of abdominal surgeries was admitted to our hospital with a chief complaint of recent-onset vomiting lasting two days. The patient had a history of type 2 diabetes mellitus and reported a history of chronic abdominal discomfort. Abdominal computed tomography (CT) revealed a mechanical obstruction with a high-density mass in the third portion of the duodenum (Figure 1).

**Figure 2:**
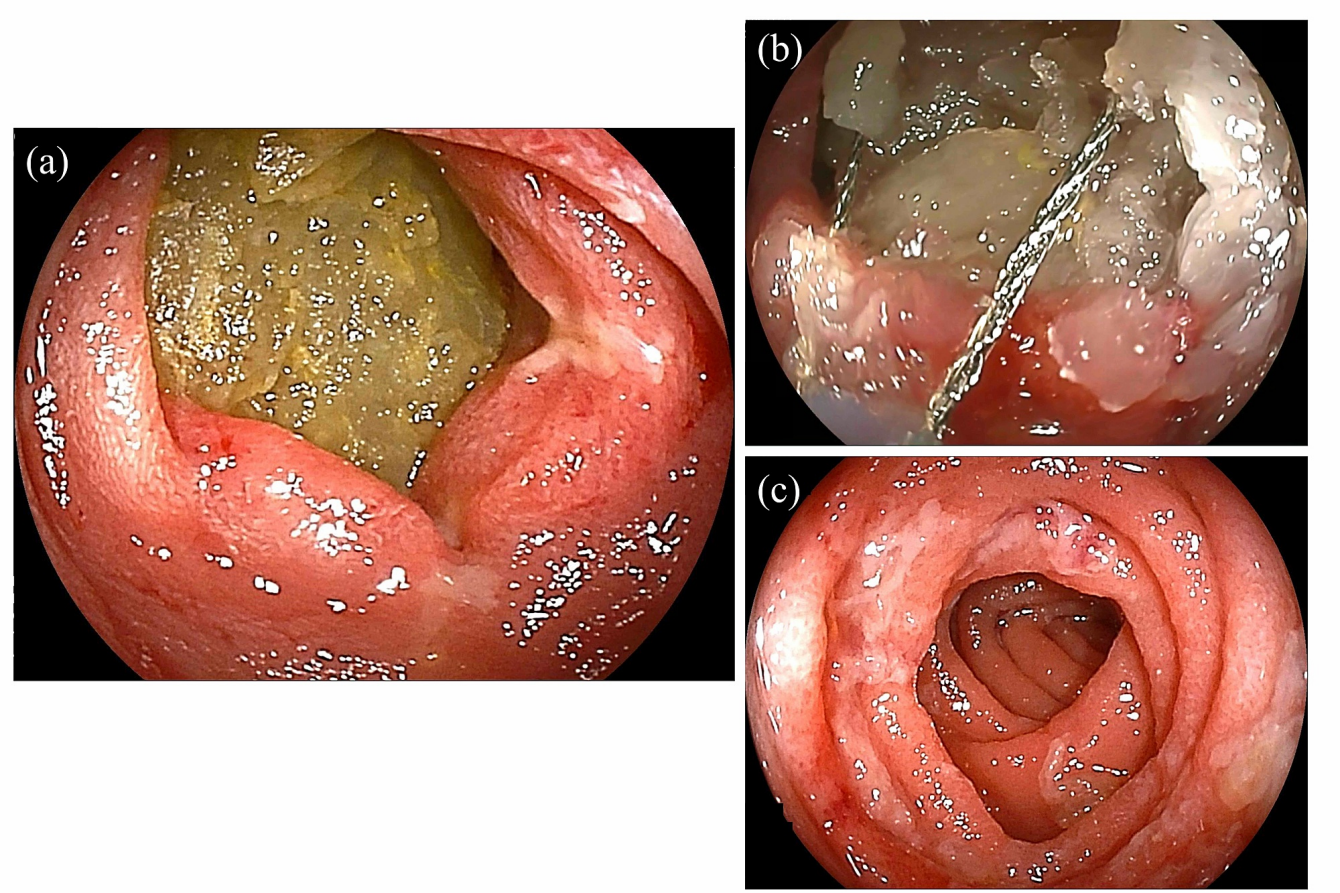
Endoscopic finding of the duodenum (a) Food complex impacted in the lumen (b) Removal of the surface of the mass using an endoscopic snare (c) Re-opening of the duodenal tract

**Figure 3:**
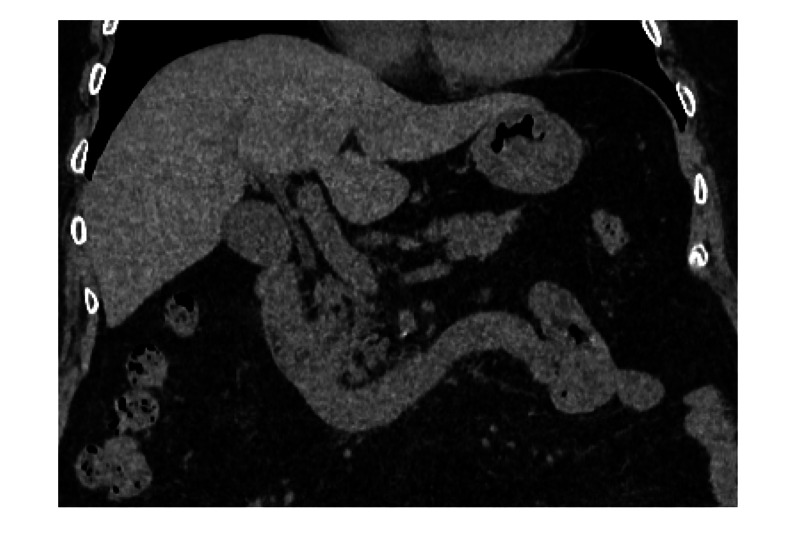
The abdominal computed tomography findings obtained the next day It shows no evidence of perforation, and the ileus had disappeared.

After draining the pooled liquid in the stomach using a nasogastric tube, double-balloon enteroscopy (DBE) was used to confirm the etiology because the mass was not located within the reach of the esophagogastroduodenoscope. A food complex with a jelly-like appearance, which was not suspected to be a neoplasm or a gallstone, was impacted at the site (Figure 2a). The food mass was tightly fixed, immobile, and was too fragile to be grasped using forceps. Instead of surgery, we attempted an endoscopic removal of the mass using the SnareMaster (Olympus, Tokyo, Japan) (Figure 2b). We performed fragmentation without energization for 1 hour. The food complex was elaborately fragmented into small pieces to avoid impacting the distal small bowel. Thereafter, the duodenal tract was reopened, demonstrating the success of endoscopic disimpaction in this case (Figure 2c). The scope was advanced to the jejunum beyond the Treitz’ ligament without any resistance. In addition to this, no stricture was identified, which was verified using the radiological contrast test during DBE. Ulcers and surrounding mucosal edema observed at the site were considered to have developed from the ischemic injury caused by the pressure of the impacted food complex, which had the same etiology as that of solitary rectal ulcers [7]. Biopsy specimens obtained at the site showed no specific findings, and Crohn’s disease, eosinophilic enteropathy, and amyloid deposits were not suspected. The peristalsis of the area appeared relatively weak . An abdominal CT scan obtained the next day showed no evidence of perforation, and the ileus had disappeared (Figure 3). The patient was discharged 7 days after treatment without any adverse events.

**Figure 4:**
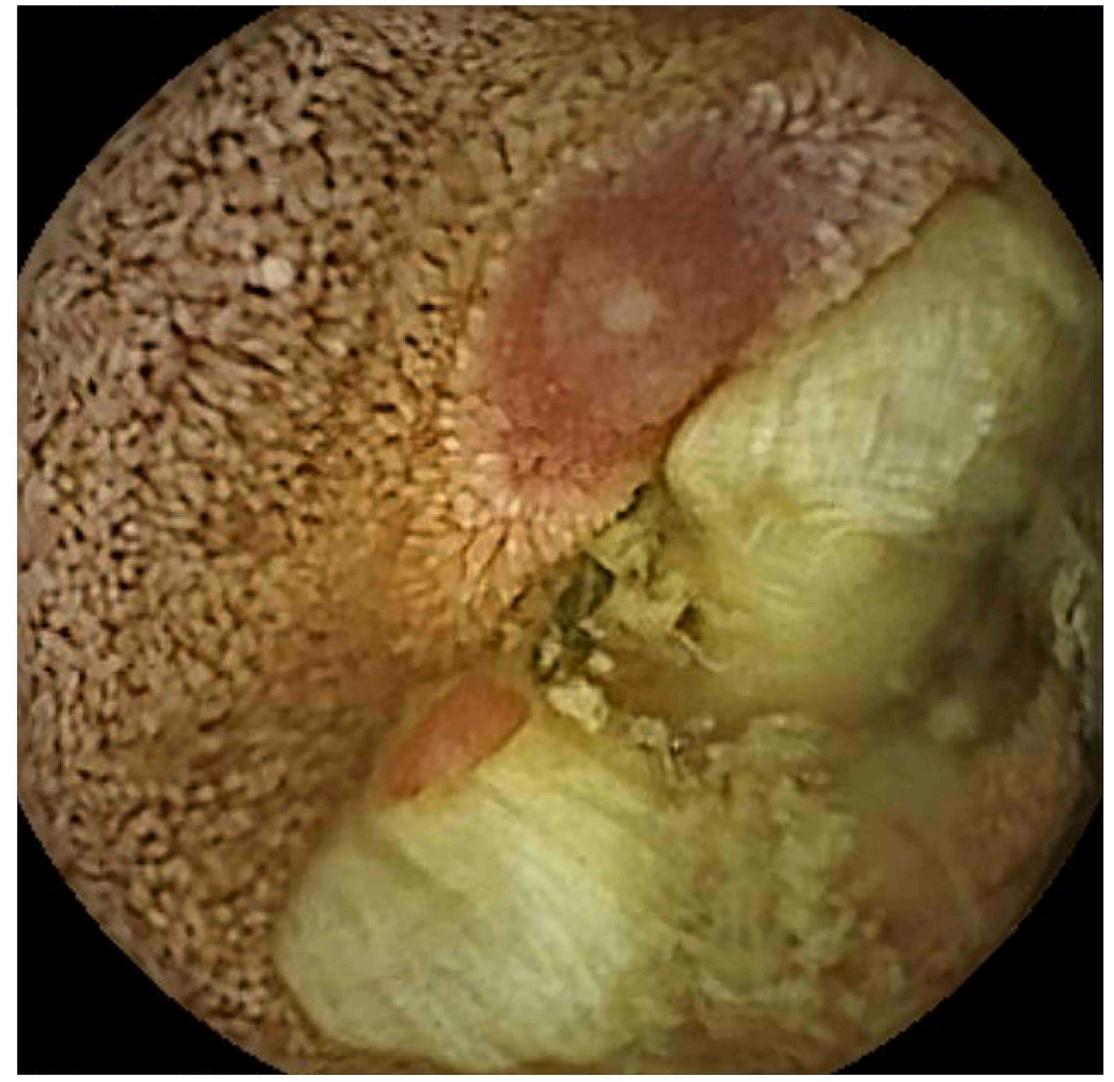
Capsule endoscopic examination Delayed passage of a food residue in the third portion of the duodenum.

**Figure 5:**
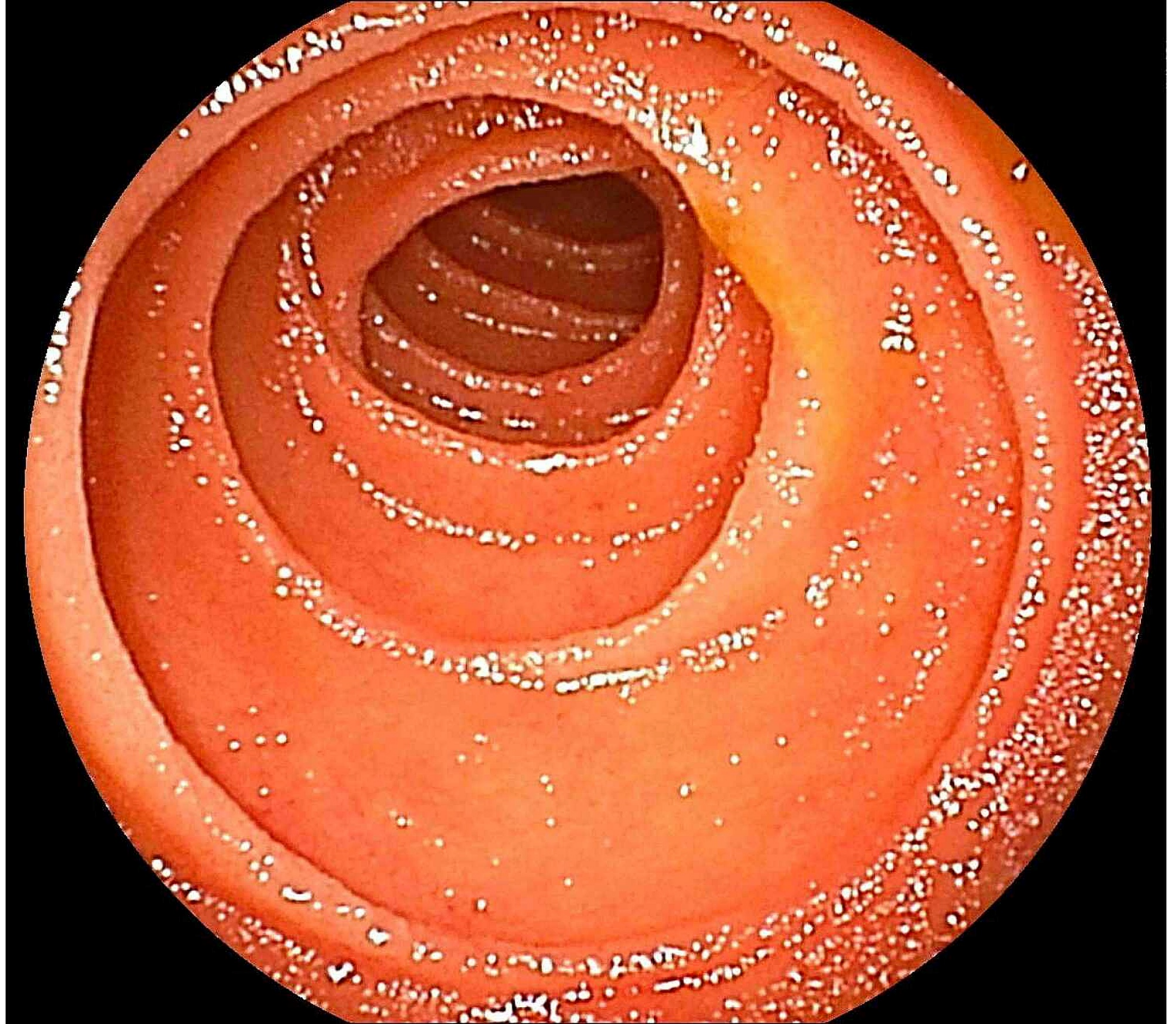
Double-balloon enteroscopic finding 6 months after treatment Neither ulcer nor stricture is observed at the treated site.

Capsule endoscopy (CE) examination was performed one month after discharge to assess the entire small bowel. No abnormalities were noted in the jejunum or ileum. A delayed passage time of 120 minutes within the duodenum accounted for 39% of the total small bowel exploration time, and food residue was observed floating in the third portion of the duodenum, suggesting that the abnormal motility of the duodenum caused food clustering, resulting in a bowel obstruction (Figure 4). The patient had not taken non-steroidal anti-inflammatory drugs or any medication that could decrease the motility of the duodenum. Her diabetes was well controlled, as indicated by a hemoglobin A1c of 6.6%. Moreover, hypothyroidism was not observed as her thyroid-stimulating hormone (normal range, 0.34-4.0 μIU/mL) and free thyroxine 4 (normal range, 0.8-1.7 ng/mL) levels were 2.76 μIU/mL and 1.03 ng/dL respectively. She had no history of alcohol consumption. Recurrence of an obstruction was not seen after 6 months owing to the administration of the medication that increased gastroduodenal motility after the endoscopic treatment. Follow-up DBE confirmed no residual abnormality at the site (Figure 5).

## Discussion

While cases of food-derived ileus have been reported, it remains a rare condition. Fourteen cases of small bowel ileus induced by rice cakes have been reported, all of which were resolved after conservative treatment [8]. A case of jejunal ileus due to an impaction of shiitake mushrooms was reported, which was treated endoscopically using a snare to fragment the impaction [9], similar to our case. Other ileus cases induced by foreign bodies, such as gallstones, phytobezoars, and enteroliths, have also been reported [10-12]. Most of these cases required surgical treatment for disimpaction. When retrospectively reviewing our case, conservative treatment was not likely to release the obstruction due to the tight impaction of the food complex. Due of the anatomical complexity of the duodenum and a lack of evidence for neoplasm in this case, a surgical procedure would have been excessive. Therefore, endoscopic treatment was considered a reasonable option. 

The etiology of the duodenal obstruction in this case is still obscure. Instead of manometry or magnetic resonance enterography, CE was used to determine the etiology in this case after confirming the absence of a small bowel stricture using CT (e.g., we confirmed a lack of both intestinal narrowing with distension at the oral side and extensive bowel wall thickness [13]). Not only did CE reveal the food residue retention, but also the abnormal motility of the duodenum. Although CE has the same diagnostic ability for small bowel lesions as DBE [14], CE can also obtain information about the physiological activity of the small bowel. In this case, a remarkably delayed transit time through the duodenum caused food clustering at the site, which resulted in an obstruction. Functional dyspepsia (FD) and diabetic neuropathy could be etiologies of the duodenal dysmotility in our case. 

Changes in the number of intestinal cells of Cajal and myenteric neurons due to impaired mucosal integrity and low baseline impedance in the duodenum which result in abnormal gastroduodenal motility, have been reported in patients with FD [15, 16]. Alternatively, a high-fat diet and microbial alteration are thought to cause enteric diabetic neuropathy and intestinal dysmotility based on the reduction of nitrergic myenteric neurons per ganglion in the duodenum [17]. The median time of retention in the duodenum was reported to be longer in diabetic patients than in healthy volunteers (12.7 versus 8.1 minutes) [18]. 

## Conclusions

This case demonstrated that endoscopic examination may aid in the treatment of ileus, with more favorable outcomes than conservative therapy. The use of DBE could help make a decision for optimal treatment in cases of ileus. Considering the anatomical complexity of the duodenum, transluminal endoscopic treatment could be a viable option to prevent the unnecessary performance of surgery. Moreover, after resolving the ileus status, CE can be clinically useful in clarifying the etiology as it provides information regarding duodenal motility as well as intra-luminal abnormality.
